# Secondary aortoenteric fistula possibly associated with continuous physical stimulation: a case report and review of the literature

**DOI:** 10.1186/s13256-019-2003-1

**Published:** 2019-03-15

**Authors:** Hiroaki Saito, Yoshitaka Nishikawa, Jun-ichi Akahira, Hajime Yamaoka, Toru Okuzono, Toyoaki Sawano, Masaharu Tsubokura, Kazuhiro Yamaya

**Affiliations:** 1 Department of Gastroenterology, Sendai Kousei Hospital, Sendai, Miyagi Japan; 2 Department of Internal Medicine, Hirata Central Hospital, Fukushima, Ishikawa Japan; 3 Department of Health Informatics, Kyoto University School of Public Health, Kyoto, Kyoto Japan; 4 Department of Pathology, Sendai Kousei Hospital, Sendai, Miyagi Japan; 5 Sendai Gastrointestinal Endoscopy Clinic, Sendai, Miyagi Japan; 6 Department of Surgery, Minamisoma Municipal General Hospital, Minamisoma, Fukushima Japan; 7 Department of Public Health, Fukushima Medical University School of Medicine, Fukushima, Fukushima Japan; 8 Department of Cardiovascular Surgery, Sendai Kousei Hospital, Sendai, Miyagi Japan

**Keywords:** Aortoenteric fistula, Abdominal aortic aneurysm, Intestinal bleeding, Herald bleeding

## Abstract

**Background:**

Secondary aortoenteric fistula is a rare but fatal complication after reconstructive surgery for an aortic aneurysm characterized by abdominal pain, fever, hematochezia, and hematemesis, and the mortality rate is high. It has been suggested that it arises due to either continuous physical stimulation or prosthesis infection during primary surgery. We describe an aortoenteric fistula following reconstructive surgery for an abdominal aortic aneurysm together with postmortem pathological findings.

**Case presentation:**

A 59-year-old Japanese man who had undergone reconstructive surgery for an abdominal aortic aneurysm 20 months earlier presented with the chief complaint of hematochezia and malaise. Esophagogastroduodenoscopy and total colonoscopy revealed only colon diverticula with no bleeding. Contrast-enhanced computed tomography revealed gas within the aneurysm sac and adhesion between the replaced aortic graft and intestinal tract, suggesting a graft infection. After 18 days of antibiotic treatment, he suddenly went into a state of shock, with massive fresh bloody stool and hematemesis, followed by cardiac arrest. An autopsy revealed communication between the artery and the ileum through an ulcerative fistula at the suture line between the left aortic graft branch and the left common iliac artery. Pathological analysis revealed tight adherence between the arterial and intestinal walls, but no marked sign of infection around the fistula, suggesting that the fistula had arisen due to physical stimuli.

**Conclusions:**

Pathological analysis suggested that the present secondary aortoenteric fistula arose due to physical stimuli. This reaffirms the importance of keeping reconstructed aortas isolated from the intestine after abdominal aortic aneurysm surgery.

## Background

A secondary aortoenteric fistula (SAEF) is an abnormal connection between the aorta and gastrointestinal tract resulting from reconstructive surgery for an abdominal aortic aneurysm (AAA), including open repair surgery and endovascular treatment. Although rare (incidence rate, 1.6 to 4%) [1–4], it is life-threatening and has a high mortality rate (24 to 45.8%) [5–7]. Elucidating the clinical features and improving the diagnosis of SAEF would therefore be valuable.

The mechanism underlying the pathological development of SAEFs remains unknown. Two hypotheses have been postulated. One suggests an origin in continuous stimulation due to aortic pulsation directly affecting the walls of the intestinal tract and arteries [2, 4, 7–9]. This theory is supported by the fact that most SAEFs involve the third or fourth portion of the duodenum which are compressed between the superior mesenteric artery and abdominal aorta in the retroperitoneal space [5]. The other argues an origin in a local inflammatory response due to prosthesis infection during primary surgery [5, 7–10]. Several species of bacteria not usually identified in the intestine, such as *Staphylococcus*, have been detected in aortic prostheses in cases of SAEF, which supports this hypothesis.

Here, we describe an aortoenteric fistula following reconstructive surgery for an AAA and discuss the implications of postmortem pathological analysis. We believe the findings further our understanding of the underlying mechanism of SAEFs, which could be useful in deciding a preventive strategy.

Written informed consent was obtained from the patient’s family for publication of this case.

## Case presentation

A 59-year-old Japanese man presented to our hospital with the chief complaint of hematochezia and malaise. On the day of admission and 10 days earlier, he had produced a fresh bloody stool. He had undergone open surgery with a bifurcated graft for an AAA 20 months earlier. The course was uneventful, with no remarkable findings on computed tomography (CT) at 6 and 18 months postoperatively. An abdominal examination at our hospital revealed nothing remarkable and no tenderness. His blood pressure was 122/75 mmHg; heart rate, 86/minute; body temperature, 36.6 °C; breathing, 16 per minute; and hemoglobin level, 9.0 g/dL.

**Fig. 1:**
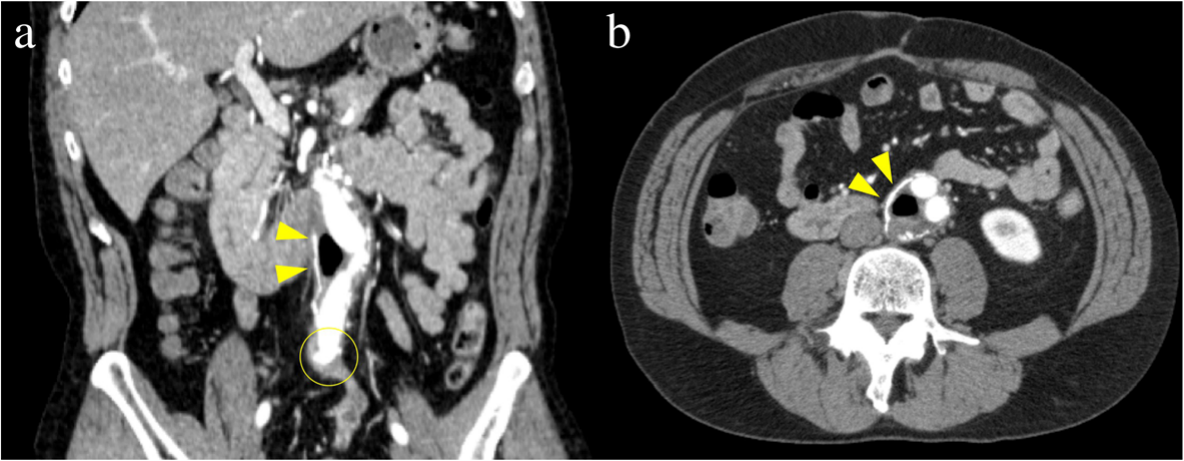
Contrast-enhanced computer tomography (coronary (**a**) and axial (**b**) view) revealed gas within aneurysm sac (*yellow arrowhead*), and attachment of ileum to anastomosis between left branch of graft and left common iliac artery (*yellow circle*)

Esophagogastroduodenoscopy and total colonoscopy revealed only colon diverticula and no bleeding. Contrast-enhanced CT revealed gas within the aneurysm sac (Fig. 1a, b, yellow arrowhead) and adhesion between the graft and intestinal tract in three areas: the ileum had attached to the anastomosis between the left branch of the graft and left common iliac artery (Fig. 1a, yellow circle); the jejunum to the middle of the graft body; and the duodenum to the anastomosis between the aorta and the proximal graft. *Enterococcus faecium* was isolated from blood culture, suggesting communication between the intestinal tract and aorta at the attached sites, possibly due to infection of the graft. His vital signs were stable, so surgery was scheduled to take place after antibiotic treatment. After admission, he produced another fresh bloody stool, but bleeding ceased immediately. At 18 days after the second fresh bloody stool, however, he suddenly went into a state of shock, with massive fresh bloody stool and hematemesis, followed by cardiac arrest. Despite intensive cardiopulmonary resuscitation, he died from hemorrhagic shock.

**Fig. 2:**
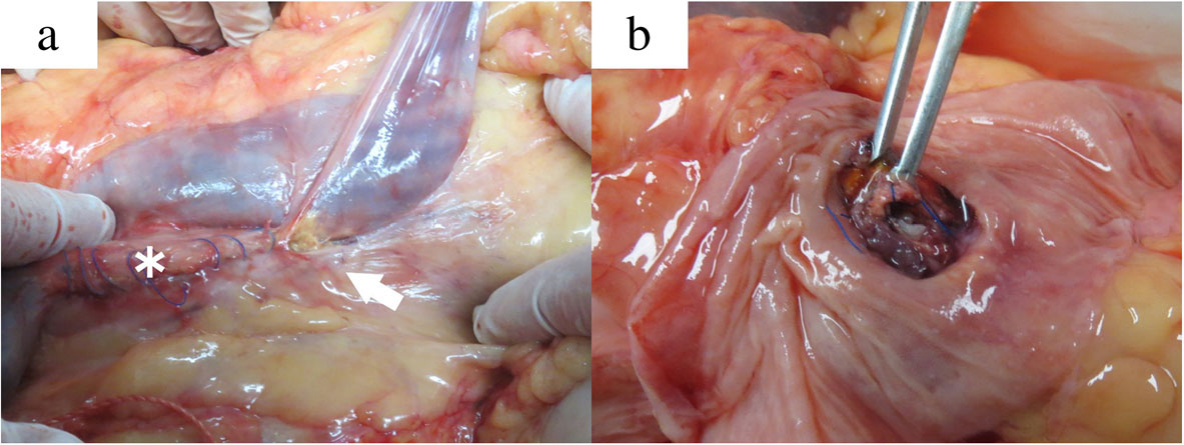
Image at autopsy. Adhesion of the distal ileum to the anastomosis between the left branch of the graft and the left common iliac artery (*arrow*), and the postoperative aneurysm sac on the head side (*asterisk*) (**a**), with a small hole at the aortic anastomosis in dissected ileum (**b**)

**Fig. 3:**
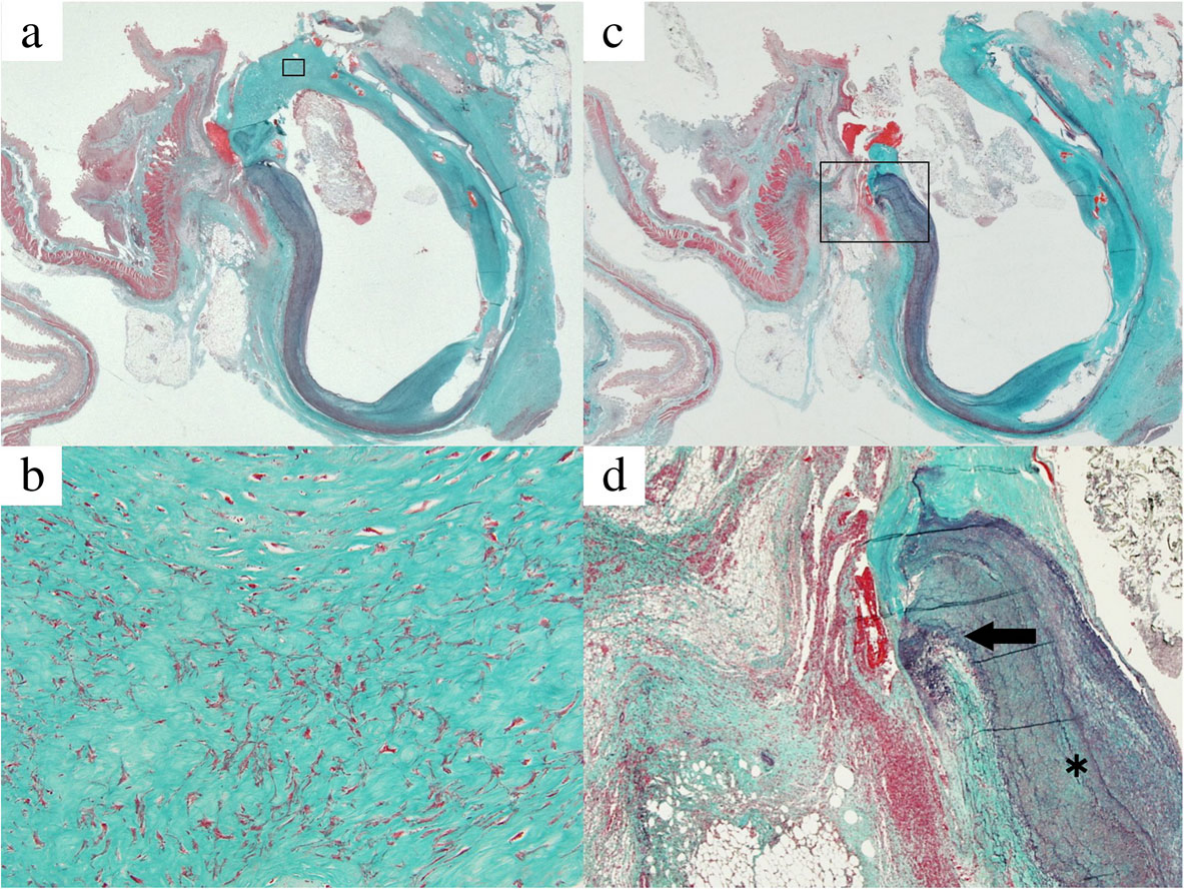
Histology of left common iliac artery by Elastica van Gieson stain revealed fibrotic and thickened intima of anastomosed artery exposed to intestinal lumen at edge of fistula (**a** *whole*, magnification × 1; **b** *square*, magnification × 400). Histology of fistula revealed firm adhesion (*arrow*) between serosa of intestinal tract and adventitia of artery (*asterisk*) (**c** *whole*, magnification × 1; **d** *square*, magnification × 40)

An autopsy performed with written consent from the family revealed an ulcerative fistula in the distal ileum that adhered to the anastomosis between the left branch of the graft and the left common iliac artery (Fig. 2a), with a small hole at the aortic anastomosis (Fig. 2b). Arterial structure was destroyed at the anastomotic site. There was fibrous thickening of the arterial wall (Fig. 3a, b) and the external elastic lamina had disappeared. The serosa of the small intestine and adventitia of the artery were firmly adhered (Fig. 3c, d). There was no marked sign of infection, such as inflammatory cells or phagocytosis, even around the fistula.

## Discussion

SAEFs are a rare but life-threatening complication after aortic aneurysm reconstruction [1–3, 11]. Despite the importance of establishing the underlying mechanism of fistula formation, their etiology remains unclear. It has been suggested that either chronic infection of the graft or physical stimulation, such as from aortic pulsation pressure, contributes to their formation, which is usually determined based solely on the clinical course or surgical findings [12–19]. To the best of our knowledge, only 25 case reports on SAEFs have been published (see Table 1). Of these, only six discuss the possible mechanism of fistula formation and the results of pathological examination [20–25]; and only two of these reports include detailed pathological images [20, 25]. Therefore, the precise mechanism underlying fistula formation remains to be clarified pathologically.

**Table 1:** Summary of published case reports describing secondary aortoenteric fistula after both open surgery and endovascular aortic reconstruction for abdominal aortic aneurysm

	Date	Authors and reference number	Age, sex	Clinical presentation	Initial treatment for aneurysm	Period^†^	Treatment for SAEF	Outcome	Pathological examination^‡^	Estimated aetiology^§^
1	2018	Arworn *et al*. [20]	42 M	Hematemesis, melena	EVAR	1 yr 1 m	Aortic reconstruction, bowel repair	Alive at 9 months	+	Mechanical factor
2	2018	Alfawaz *et al*. [28]	62 M	Malaise, fever	Open surgery	7 yr	Aortic reconstruction, bowel repair	Alive at 7 days	–	Unclear
3	2017	Dickfos *et al*. [19]	75 M	Abdominal pain, fevers	EVAR	1 yr 2 m	Aortic reconstruction	Unclear	–	Infection
4	2016	Kadhim *et al*. [21]	66 M	Confusion, fever	EVAR	15 yr	Bowel repair, debridement	Alive at 12 months	+	Infection
5	2017	Guevara-Noriega *et al*. [18]	65 M	Malaise	Open surgery	1 yr 10 m	Aortic reconstruction, bowel repair	Alive at 2 years	–	Infection
6	2014	Zaki *et al*. [17]	75 M	Abdominal pain, hematemesis	EVAR	2 yr 6 m	Aortic reconstruction, bowel repair	Died	–	Infection
7	2013	Jamal *et al*.[29]	60 F	Hematemesis, melena	Open surgery	6 yr	Surgery	Died	–	Unclear
8	2009	Grassia *et al*. [30]	72 M	Hematochezia	Open surgery	8 yr	Aortic reconstruction	Alive at 2 weeks	–	Unclear
9	2009	McAloon *et al*. [31]	73 M	Lethargy, melena	Open surgery	10 yr	None^*^	Died	–	Unclear
10	2008	Hara *et al*. [16]	84 M	Hematochezia	Open surgery	14 yr	Aortic reconstruction, bowel repair	Alive at 6 months	–	Recurrent aneurysm
11	2008	Geraci *et al*. [15]	59 M	Dyspepsia, vomiting	Open surgery	5 yr	Bowel repair	Alive at 6 months	–	Mechanical factor
12	2008	Bognar *et al*. [22]	67 M	Rectal bleeding	Open surgery	4 yr	Aortic reconstruction, bowel repair	Alive at 24 days	+	Mechanical factor
13	2007	Brountzos *et al*. [32]	85 M	Gastrointestinal bleeding	Open surgery	10 yr	EVAR	Alive at 1 year	–	Unclear
14	2006	Heidemann *et al*. [33]	52 M	Hematochezia, hematemesis	Open surgery	6 m	Aortic reconstruction, bowel repair	Alive at 8 months	–	Unclear
15	2007	Tsunekawa *et al*. [23]	75 M	Fever, malaise	Open surgery	15 yr	Aortic reconstruction, bowel repair	Alive at 1 month	+	Infection
16	2006	Maternini *et al*. [14]	73 M	Melena	Open surgery	15 yr	EVAR	Alive	–	Unclear
17	2005	Mundal *et al*. [24]	82 F	Hematemesis	Open surgery	17 yr	Surgery	Died	+	Unclear
18	2004	French *et al*. [13]	68 F	Hematemesis	EVAR	1 yr	Aortic reconstruction, bowel repair, debridement	Died	–	Infection
19	2002	Tomlinson *et al*. [34]	90 M	Melena, abdominal pain	Open surgery	5 yr	EVAR	Alive at 14 months	–	Unclear
20	2000	Makar *et al*. [35]	70 M	Epigastric discomfort	EVAR	4 m	Antibiotics	Died	–	Unclear
21	1999	Karacagil *et al*. [36]	70 F	Melena, fever	Open surgery	14 yr	Aortic reconstruction, bowel repair	Alive at 2 years	–	Unclear
22	1993	Neergaard *et al*. [37]	69 M	Hematemesis, melena	Open surgery	8 yr	Aortic reconstruction, bowel repair	Alive at 4 months	–	Unclear
23	2018	Jiang *et al*. [38]	85 M	Melena, tiredness, dizziness, fever	EVAR	2 yr 4 m	Aortic reconstruction, bowel repair, debridement	Alive at 15 months	–	Unclear
24	2017	Hansen *et al*. [12]	75 F	Melena	Open surgery	2 yr	Open surgery + EVAR	Alive at 12 years	–	Infection
25	1998	Yabu *et al*. [25]	77 M	Dyspnea	Open surgery	10 yr	None^*^	Died	+	Mechanical factor

*EVAR* endovascular aortic reconstruction, *F* female, *m* month, *M* male, *SAEF* secondary aortoenteric fistula, *yr* year

†period after abdominal aortic aneurysm treatment

‡whether pathological examination was performed or not

§possible cause suggested by authors of the report

*patient died immediately before any treatment

The present autopsy findings suggested that physical stimuli following postoperative adhesion and attachment between the iliac artery and small bowel had contributed to the formation of the fistula, although chronic infection of the graft could not be excluded completely. Despite fibrillar change in the serous membrane and muscularis propria layer of the intestine, inflammatory cell infiltration was sparse at the edge of the fistula. Moreover, there was no sign of phagocytosis, indicating no destructive infection of the graft. The arterial wall exhibited fibrous thickening, however, possibly due to continuous physical stimulation. These pathological findings and the clinical course suggested that the persistent pulsation of the aorta had physically degraded the attached intestinal wall and aortic membrane, inducing erosion of the bowel tract. Fatal bleeding appears to have occurred when the vulnerable inner membrane of the attached aorta finally ruptured.

In terms of the underlying mechanism of fistula formation, the present findings reaffirm the importance of avoiding adhesion between the intestinal wall and the anastomosis of the graft due to insufficient isolation of the latter [26]. Interposing native tissue between the aorta and the intestinal tract at primary surgery has been reported as effective in preventing SAEF associated with physical stimuli [1]. In the present case, the artery and intestinal tract had adhered tightly at the site of the fistula, even though the artificial graft had been covered with a longitudinally split aneurysmal wall and retroperitoneum during primary surgery. This particular fistula involved both the ileum and the distal suture line of the graft, a relatively rare site for an SAEF. The ileum might have become trapped by adhering to the closing line of the retroperitoneum after primary surgery, resulting in tight contact with the distal anastomosis of the graft. This suggests that keeping a graft isolated from the intestinal wall in the usual fashion during surgery may only be effective up to a certain point, and that further adjuvant maneuvering may be necessary to maintain postoperative isolation. Adhesive barriers could be applied to cover the closing line of the retroperitoneum.

Clinically, SAEFs present in a variety of ways, which makes a prompt diagnosis challenging [3, 8]. Whereas gastrointestinal bleeding occurs in most such cases, sepsis occurs in only approximately half on initial presentation [5, 8, 27], which is possibly because such symptoms are unlikely if the fistula was formed mainly due to mechanical stimuli. Infection might occur later, however, through contact with intestinal bacteria. Therefore, an SAEF should be suspected in cases of bloody stool or hematemesis after AAA reconstruction, even in the absence of septic symptoms. If an SAEF is the suspected cause of bloody stool, immediate exploratory surgery should be considered to prevent potentially catastrophic developments, even if the general status of the patient is stable.

## Conclusions

This case report described gastrointestinal bleeding due to an SAEF. Physical stimuli were associated with its formation due to adhesion between the aorta and the intestinal wall after AAA reconstruction, indicating the importance of keeping a reconstructed aorta isolated from the intestine. Clinicians should suspect SAEF in patients with bloody stool after aneurysm surgery.

## Abbreviations

AAA: Abdominal aortic aneurysm
CT: Computed tomography
SAEF: Secondary aortoenteric fistula

## References

[1] Wilson WR, Bower TC, Creager MA, Amin-Hanjani S, O’Gara PT, Lockhart PB, Darouiche RO, Ramlawi B, Derdeyn CP, Bolger AF, *et al.* Vascular Graft Infections, Mycotic Aneurysms, and Endovascular Infections: A Scientific Statement From the American Heart Association. Circulation. 2016;134(20):e412–60. 10.1161/CIR.0000000000000457 27737955

[2] Champion MC, Sullivan S, Coles J, Goldbach M, Watson WC. Aortoenteric fistula. Incidence, presentation recognition, and management. Ann Surg. 1982;195(3):314–7. 10.1097/00000658-198203000-00011 PMC1352637 7059240

[3] Hallett JW Jr, Marshall DM, Petterson TM, Gray DT, Bower TC, Cherry KJ Jr, Gloviczki P, Pairolero PC. Graft-related complications after abdominal aortic aneurysm repair: reassurance from a 36-year population-based experience. J Vasc Surg. 1997;25(2):277–84. discussion 285-276 10.1016/s0741-5214(97)70349-5 9052562

[4] Chenu C, Marcheix B, Barcelo C, Rousseau H. Aorto-enteric fistula after endovascular abdominal aortic aneurysm repair: case report and review. Eur J Vasc Endovasc Surg. 2009;37(4):401–6. 10.1016/j.ejvs.2008.11.037 19211278

[5] Malik MU, Ucbilek E, Sherwal AS. Critical gastrointestinal bleed due to secondary aortoenteric fistula. J Community Hosp Intern Med Perspect. 2015;5(6):29677. 10.3402/jchimp.v5.29677 PMC4677592 26653698

[6] Gnus J, Ferenc S, Koscielna M, Paprocka-Borowicz M, Dawidczyk P, Dziewiszek M, Witkiewicz W. Secondary Aortoenteric Fistula After Abdominal Aortic Graft Implementation in Our Own Material. Adv Clin Exp Med. 2016;25(6):1265–71. 10.17219/acem/66621 28028982

[7] Armstrong PA, Back MR, Wilson JS, Shames ML, Johnson BL, Bandyk DF. Improved outcomes in the recent management of secondary aortoenteric fistula. J Vasc Surg. 2005;42(4):660–6. 10.1016/j.jvs.2005.06.020 16242551

[8] Bergqvist D, Bjorck M. Secondary arterioenteric fistulation-a systematic literature analysis. Eur J Vasc Endovasc Surg. 2009;37(1):31–42. 10.1016/j.ejvs.2008.09.023 19004648

[9] Perdue GD Jr, Smith RB 3rd, Ansley JD, Costantino MJ. Impending aortoenteric hemorrhage: the effect of early recognition on improved outcome. Ann Surg. 1980;192(2):237–43. 10.1097/00000658-198008000-00019 PMC1344860 6967716

[10] Takeda Y, Daimon M, Katsumata T, Morita H, and Ishizaka N. Repetitive complications after prosthetic graft for inflammatory aortic aneurysm. SAGE Open Med Case Rep. 10.1177/2050313X13513230. PMC4857269 27489635

[11] Tagowski M, Vieweg H, Wissgott C, Andresen R. Aortoenteric fistula as a complication of open reconstruction and endovascular repair of abdominal aorta. Radiol Res Pract. 2014;2014:383159. 10.1155/2014/383159 PMC4180205 25302119

[12] Hansen BA, Amundsen S, Reikvam H, Wendelbo O, Pedersen G. Non-curative surgery for aortoenteric fistula. J Surg Case Rep. 2017;2017(8):rjx153. 10.1093/jscr/rjx153 PMC5570076 28852462

[13] French JR, Simring DV, Merrett N, Thursby P. Aorto-enteric fistula following endoluminal abdominal aortic aneurysm repair. ANZ J Surg. 2004;74(5):397–9. 10.1111/j.1445-1433.2004.03000.x 15144275

[14] Maternini M, Tozzi P, Vuilleumier H, Von Segesser LK. Intra vascular ultra sound: one more tool to diagnose aorto-duodenal fistula. Eur J Vasc Endovasc Surg. 2006;32(5):542–4. 10.1016/j.ejvs.2006.05.011 16861015

[15] Geraci G, Pisello F, Li Volsi F, Facella T, Platia L, Modica G, Sciume C. Secondary aortoduodenal fistula. World J Gastroenterol. 2008;14(3):484–6. 10.3748/wjg.14.484 PMC2679141 18200675

[16] Hara H, Shinji A, Mukawa K, Takayama M, Okiyama W, Yamamura N, Oguchi H. Internal iliac artery aneurysm rupture with aorto-enteric fistula after reconstruction of abdominal aortic aneurysm: report of a case. Nihon Shokakibyo Gakkai Zasshi. 2008;105(2):221–7. 18250593

[17] Zaki M, Tawfick W, Alawy M, ElKassaby M, Hynes N, Sultan S. Secondary aortoduodenal fistula following endovascular repair of inflammatory abdominal aortic aneurysm due to *Streptococcus anginosus* infection: A case report and literature review. Int J Surg Case Rep. 2014;5(10):710–3. 10.1016/j.ijscr.2013.10.016 PMC4189064 25201477

[18] Guevara-Noriega KA, Velescu A, Zaffalon-Espinal DT, Mateos-Torres E, Roig-Santamaria L, Clara-Velasco A. Aorto-bifermoral graft infection due to *Candida parapsilosis*. An unusual pathogen. Cirugia y Cirujanos. 2017;85(3):234–9. 10.1016/j.circir.2016.01.002 27039287

[19] Dickfos M, Garnham K, Jenkins J. *Salmonella typhimurium* Infected Abdominal Aortic Aneurysm Endovascular Repair with Secondary Aortoenteric Fistula Formation. S Afr J Surg. 2017;55(3):77.

[20] Arworn S, Orrapin S, Chakrabandhu B, Reanpang T, Settakorn J, Laohapensang K. Aorto-enteric Fistula After Endovascular Abdominal Aortic Aneurysm Repair for Behcet’s Disease Patient: A Case Report. EJVES Short Rep. 2018;39:54–7. 10.1016/j.ejvssr.2018.05.004 PMC6019691 29988290

[21] Kadhim MMK, Rasmussen JBG, Eiberg JP. Aorto-enteric Fistula 15 Years After Uncomplicated Endovascular Aortic Repair with Unforeseen Onset of Endocarditis. EJVES Short Rep. 2016;31(2405–6553 (Print)):16–8. 10.1016/j.ejvssr.2016.03.004 PMC5573115 28856303

[22] Bognar G, Sugar I, Sipos P, Ledniczky G, Laczko A, Ondrejka P. Secondary iliac-enteric fistula to the sigmoid colon complicated with entero-grafto-cutaneous fistula. Case Rep Gastroenterol. 2008;2(1):138–43. 10.1159/000121470 PMC3075182 21490854

[23] Tsunekawa T, Ogino H, Minatoya K, Matsuda H, Sasaki H, Fukuchi K. Masked prosthetic graft to sigmoid colon fistula diagnosed by 18-fluorodeoxyglucose positron emission tomography. Eur J Vasc Endovasc Surg. 2007;33(2):187–9. 10.1016/j.ejvs.2006.06.010 16904347

[24] Mundal L, Ignjatovic D, Vage V, Asmussen I, Sund S. A woman with hemorrhagic shock. Tidsskr Nor Laegeforen. 2005;125(13):1833–4. 16025570

[25] Yabu M, Himeno S, Kanayama Y, Furubayashi T, Kiriyama K, Nagasawa Y, Takakura R, Katata T, Iwao N, Orino A. Secondary aortoduodenal fistula complicating aortic grafting, as a cause of intermittent chronic intestinal bleeding. Intern Med. 1998;37(1):47–50. 10.2169/internalmedicine.37.47 9510399

[26] Connolly JE, Kwaan JH, McCart PM, Brownell DA, Levine EF. Aortoenteric fistula. Ann Surg. 1981;194(4):402–12. 10.1097/00000658-198110000-00004 PMC1345313 7283503

[27] Biro G, Szabo G, Fehervari M, Munch Z, Szeberin Z, Acsady G. Late outcome following open surgical management of secondary aortoenteric fistula. Langenbeck’s Arch Surg. 2011;396(8):1221–9. 10.1007/s00423-011-0807-6 21604007

[28] Alfawaz A, Tashiro J, Sleeman D, Jones K, Rey J. Total retroperitoneal approach to aortic reconstruction: A novel technique for aorto-enteric fistulae and graft infections. SAGE Open Med Case Rep. 2018;6(2050-313X (Print)) 10.1177/2050313X18760467. PMC5833235 29511543

[29] Jamal K, Shaunak S, Kalsi S, Nehra D. Secondary aorto-enteric fistula presenting over a 2-month period with recurrent gastrointestinal bleeding. BMJ Case Rep. 2013;2013 10.1136/bcr-2012-008070. PMC3644905 23592810

[30] Grassia R, Staiano T, Iiritano E, Bianchi G, Dizioli P, Baratta V, Buffoli F. Gastrointestinal hemorrhage caused by secondary aorto-duodenal fistula: a case report. Eur Rev Med Pharmacol Sci. 2009;13(2):147–50. 19499851

[31] McAloon CJ, Leong WB, Garg R, Narendran P. Secondary aorto-enteric fistula: a case report and review of literature. BMJ Case Rep. 2009;2009(1757-790X (Electronic)) 10.1136/bcr.08.2008.0721. PMC3028186 21686661

[32] Brountzos EN, Vasdekis S, Kostopanagiotou G, Danias N, Alexopoulou E, Petropoulou K, Gouliamos A, Perros G. Endovascular treatment of a bleeding secondary aorto-enteric fistula. A case report with 1-year follow-up. Cardiovasc Intervent Radiol. 2007;30(5):1037–41. 10.1007/s00270-007-9099-1 17546398

[33] Heidemann J, Domagk D, Wessling J, Domschke W, Kucharzik TF. Recurrent obscure gastrointestinal bleeding caused by aorto-enteric fistula. Z Gastroenterol. 2006;44(9):981–4. 10.1055/s-2006-926740 16981071

[34] Tomlinson MA, Gold B, Thomas MH, Browning NG. Endovascular stent graft repair of a recurrent aorto-enteric fistula. Eur J Vasc Endovasc Surg. 2002;24(5):459–61. 10.1053/ejvs.2002.1720 12435349

[35] Makar R, Reid J, Pherwani AD, Johnston LC, Hannon RJ, Lee B, Soong CV. Aorto-enteric fistula following endovascular repair of abdominal aortic aneurysm. Eur J Vasc Endovasc Surg. 2000;20(6):588–90. 10.1053/ejvs.2000.1247 11136600

[36] Karacagil S, Thelin S, Grewal P, Bergqvist D. Type IV thoraco-abdominal aortic aneurysm complicated by an aorto-enteric fistula due to previous infrarenal aortic graft. Eur J Vasc Endovasc Surg. 1999;17(3):268–70. 10.1053/ejvs.1998.0756 10092906

[37] Neergaard K, Mantoni M, Andersen L. Aorto-enteric fistula: unusual CT appearance. Eur J Radiol. 1993;16(3):213–4. 10.1016/0720-048x(93)90075-x 8508838

[38] Jiang C, Chen X, Li J, Li H. A case report of successful treatment of secondary aortoenteric fistula complicated with gastrointestinal bleeding and retroperitoneal abscess in an elderly patient. Medicine. 2018;97(24):e11055. 10.1097/MD.0000000000011055 PMC6024772 29901606

